# Atrial fibrillation is associated with increased risk of lethal ventricular arrhythmias

**DOI:** 10.1038/s41598-021-97335-y

**Published:** 2021-09-13

**Authors:** Yun Gi Kim, Yun Young Choi, Kyung-Do Han, Kyongjin Min, Ha Young Choi, Jaemin Shim, Jong-Il Choi, Young-Hoon Kim

**Affiliations:** 1 Division of Cardiology, Department of Internal Medicine, Korea University College of Medicine and Korea University Anam Hospital, 73 Goryeodae-ro, Seongbuk-gu, Seoul, 02841 Republic of Korea; 2 Department of Statistics and Actuarial Science, Soongsil University, Seoul, Republic of Korea

**Keywords:** Cardiology, Medical research, Risk factors

## Abstract

Atrial fibrillation (AF) is associated with various major adverse cardiac events such as ischemic stroke, heart failure, and increased overall mortality. However, its association with lethal ventricular arrhythmias such as ventricular tachycardia (VT), ventricular flutter (VFL), and ventricular fibrillation (VF) is controversial. We conducted this study to determine whether AF can increase the risk of VT, VFL, and VF. We utilized the Korean National Health Insurance Service database for this nationwide population-based study. This study enrolled people who underwent a nationwide health screen in 2009 for whom clinical follow-up data were available until December 2018. Primary outcome endpoint was the occurrence of VT, VFL, or VF in people who were and were not diagnosed with new-onset AF in 2009. We analyzed a total of 9,751,705 people. In 2009, 12,689 people were diagnosed with new-onset AF (AF group). The incidence (events per 1000 person-years of follow-up) of VT, VFL, and VF was 2.472 and 0.282 in the AF and non-AF groups, respectively. After adjustment for covariates, new-onset AF was associated with 4.6-fold increased risk (p < 0.001) of VT, VFL, and VF over 10 years of follow-up. The risk of VT, VFL, and VF was even higher if identification of AF was based on intensified criteria (≥ 2 outpatient records or ≥ 1 inpatient record; hazard ratio = 5.221; p < 0.001). In conclusion, the incidence of VT, VFL, and VF was significantly increased in people with new-onset AF. The potential risk of suffering lethal ventricular arrhythmia in people with AF should be considered in clinical practice.

## Introduction

Atrial fibrillation (AF) is one of the most common supraventricular tachyarrhythmias, affecting 1–2% of the general population, and is associated with a significantly increased risk of adverse cardiac events^1–4^. Overall mortality in AF patients is significantly higher compared with that of the general population^5–7^. The underlying causes of the increased mortality are malignancy (23.1%), infection (17.3%), heart failure (14.5%), and stroke (6.5%) in a study based on a Japanese community AF cohort^8^. Administrative data from Germany suggest that accompanying comorbidities play an important role in the increased mortality among AF patients^9^. Although non-cardiac causes are responsible for a large proportion of mortality among AF patients, right- and left-sided heart failure, syncope, side effects of antiarrhythmic drugs, myocardial infarction, and ischemic stroke are important cardiac causes of death. Unlike the aforementioned cardiac events, the risk of lethal ventricular arrhythmias such as ventricular tachycardia (VT) and ventricular fibrillation (VF) in AF patients is not fully understood. In real-world clinical practice, AF and ventricular arrhythmia often coexist; a prior case-controlled, cross-sectional study demonstrated that the prevalence of AF was threefold higher in patients who experienced out of hospital cardiac arrest due to VF^10^. The study was limited by cross-sectional design and could not demonstrate a chronological association between AF and VF.

Atrial fibrillation is often caused by genetic mutations in ion-channels or cardiomyopathy-related genetic variants^11^. It is not surprising that such genetic variations also affect the ventricle and cause lethal ventricular arrhythmias. Furthermore, a rapid ventricular rate can stimulate the ventricle, similar to rapid ventricular pacing which can induce VT or VF during electrophysiology studies. Decreased coronary perfusion due to rapid ventricular rate might provoke ventricular arrhythmias if the patient already has tachycardia-induced cardiomyopathy or significant coronary artery stenosis. We aimed to investigate whether AF is associated with increased risk of lethal ventricular arrhythmias using Korean National Health Insurance Service (K-NHIS) data. Utilizing a prospectively collected large-scale database, we examined the chronological association between AF and ventricular arrhythmia.

## Methods

### Patients

The K-NHIS database was used to conduct this study. The people in Republic of Korea are mandatory subscribers of the K-NHIS, which is an exclusive single medical insurance system managed by the government. Medical data stored in the K-NHIS database represents the entire population of Korea. Regular health screenings are offered to all subscribers of K-NHIS and include a health questionnaire including physical activity, alcohol, and smoking status; laboratory tests such as complete blood cell counts, lipid profile, creatinine level, liver function tests, and serum fasting blood sugar; chest X-ray; and measurement of blood pressure, body weight, height, and waist circumference. Identification of people diagnosed with AF, VT, ventricular flutter (VFL), VF, and other medical conditions was conducted using inpatient and outpatient service records of the K-NHIS. A cohort consisting of people who underwent a nationwide health screening at a certain time point is a valuable research source due to the large number of subjects, sufficient follow-up duration, and vast medical records stored in the K-NHIS database. Researchers are permitted to analyze healthcare information data stored in the K-NHIS database if the study protocols are approved by the official review committee of the government (https://nhiss.nhis.or.kr/) and the institutional review board.

This study comprised people who underwent a nationwide health screening in 2009. To identify baseline medical history, data obtained from January 2002 to December 2008 were used as screening data. People who were younger than 20 years and who had prior medical records of AF, VT, VFL, VF, premature ventricular contraction (PVC), stroke, or heart failure during the screening period were excluded from this study. The exact international classification of disease (10th edition; ICD-10) codes used in this study are described in Supplementary Table S1. Clinical follow-up data were needed until December 2018. Diagnosis of VT, VFL, and VF during follow-up was counted as a main event, and diagnoses of AF after 2009 were censored. Since the K-NHIS is an exclusive mandatory medical insurance system in Korea, clinical follow-up loss is negligible. Immigrations and death events not related with VT, VFL, and VF were censored. This study was approved by the Institutional Review Board of Korea University Medicine Anam Hospital, and written informed consent was waived due to the retrospective nature of the study. The study protocol strictly adheres to the ethical guidelines of the 2008 Declaration of Helsinki and legal regulations of Republic of Korea.

### Primary outcome endpoint

Occurrence of ventricular arrhythmias, composed of VT, VFL, and VF, was the outcome endpoint of this study and was compared between people who were and were not diagnosed with new-onset AF in 2009. The incidence of VT, VFL, and VF was defined as the number of events calculated over 1000 person-years of follow-up.

### Definitions

Identification of newly diagnosed AF in 2009 was defined as ≥ one inpatient or outpatient record of ICD-10 code. People who were and were not diagnosed with new-onset AF in 2009 were defined as the AF group and non-AF group, respectively. New-onset AF was further classified into AF 1 and AF 2 by the following criteria: (i) AF 1: one outpatient record and (ii) AF 2: ≥ two outpatient records or ≥ one inpatient record based on ICD-10 codes in the K-NHIS database. One inpatient record was required for diagnosis of heart failure^12^. The diagnosis of VT, VFL, and VF was based on one inpatient or outpatient record. Previous studies have validated the robustness of these definitions^13,14^. Baseline demographics of the study cohort including age, sex, weight, height, smoking status, alcohol, and physical activity and medical comorbidities such as hypertension, diabetes mellitus, dyslipidemia, and chronic kidney disease (CKD; eGFR < 60 ml/min/1.73 m^2^) were retrieved from the K-NHIS database.

### Statistical analysis

Continuous variables were compared by Student’s t-test. Categorical variables were analyzed with the Chi-square test or Fisher’s exact test as appropriate. The cumulative incidence of outcome endpoint (a composite of VT, VFL, and VF) was depicted by Kaplan–Meier curve analysis, and the log-rank t-test was used for comparisons between the groups. Cox-regression analysis was used to calculate non-adjusted and adjusted hazard ratios (HR) with 95% confidence interval (CI). For multivariate adjustment, three models were used: (i) adjusted for age and sex; (ii) adjusted for age, sex, body mass index (BMI), smoking status, alcohol consumption status, and regular physical activity; and (iii) adjusted for age, sex, BMI, smoking status, alcohol consumption status, regular physical activity, hypertension, diabetes mellitus, and dyslipidemia. All clinical follow-up data was used for Kaplan–Meier curve analysis and Cox-regression analysis until primary event occurred or censored (due to immigrations; death events not related with VT, VFL, and VF; and diagnosis of AF after 2009). Censored data was no longer used for any analysis. The time-scale used in our Cox-regression models was time-on-study, adjusting for age as a covariate. The proportional hazards assumption was assessed by visual estimation of Kaplan–Meier curve. All Cox-regression analysis in this study showed continuous and steady divergence of cumulative incidence of lethal ventricular arrhythmia between groups suggesting assumption of proportional hazards was fulfilled. All tests were two-tailed, and p values ≤ 0.05 were considered statistically significant. All statistical analyses were performed with SAS^®^ software version 9.2 (SAS Institute, Cary, NC, USA).

## Results

### Patients

**Figure 1:**
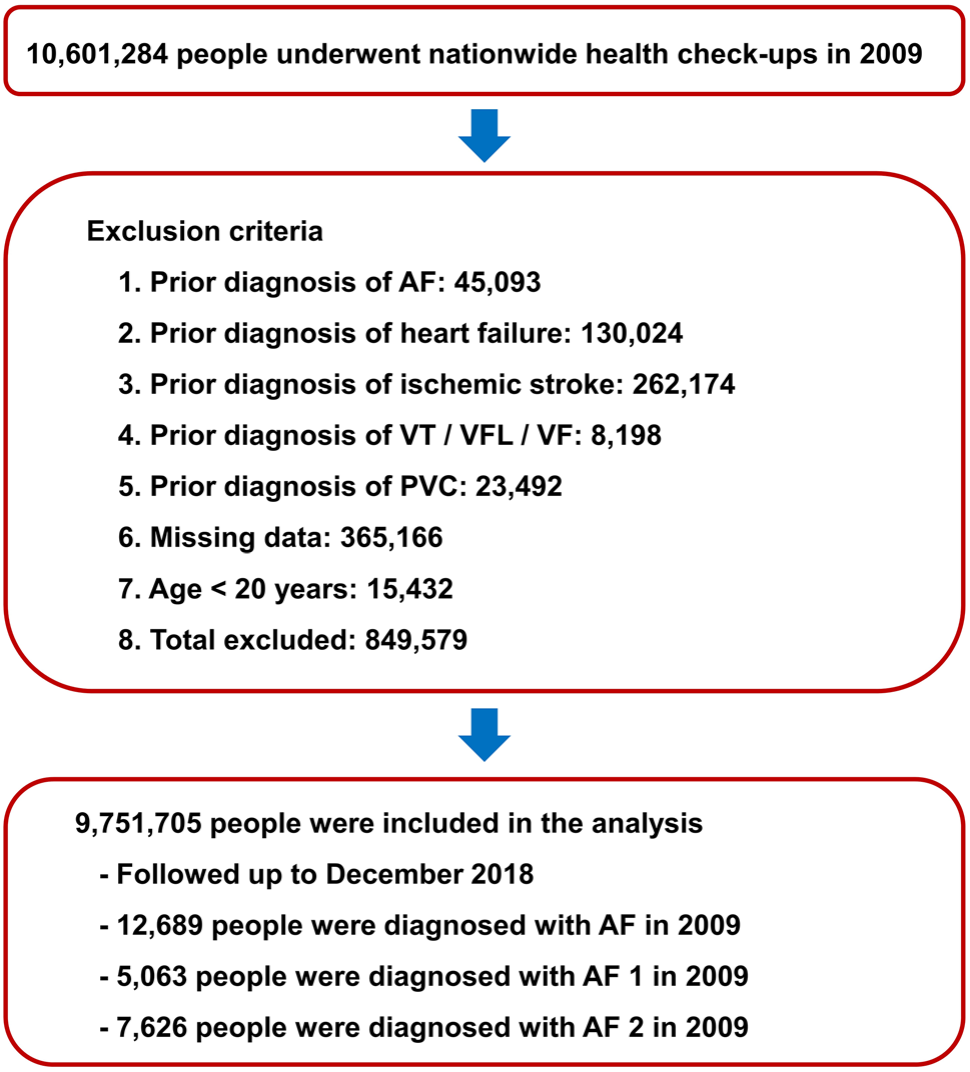
Study flow. AF: atrial fibrillation; PVC: premature ventricular contraction; VF: ventricular fibrillation; VFL: ventricular flutter; VT: ventricular tachycardia.

A total of 10,601,284 people underwent a nationwide health check-up in 2009. People were excluded from the study if they were younger than 20 years or had a prior history of AF, PVC, VT, VFL, VF, heart failure, or stroke. Finally, 9,751,705 people were included in this study. In 2009, a total of 12,689 new-onset AF cases was detected, with 5063 and 7626 cases of AF 1 (one outpatient code) and AF 2 (≥ two outpatient records or ≥ one inpatient record), respectively. Study flow is summarized in Fig. 1.

Baseline demographics of people who were and were not diagnosed with new-onset AF in 2009 are summarized in Table 1. In brief, people with new-onset AF were significantly older (46.38 ± 13.79 years vs. 56.45 ± 13.59 years; p < 0.001) and had higher prevalence of hypertension (25.09% vs. 57.44%; p < 0.001), diabetes (8.13% vs. 16.58%; p < 0.001), and dyslipidemia (17.22% vs. 30.85%; p < 0.001).

**Table 1:** Baseline demographics of the AF and non-AF groups.

	No AF	All AF (AF 1 + AF 2)	AF 1	AF 2	p value
	n = 9,739,016	n = 12,689	n = 5063	n = 7626	(no AF vs. all AF)
Male sex	5,396,129 (55.41%)	7478 (58.93%)	2823 (55.76%)	4655 (61.04%)	< 0.001
Age ≥ 65 years	1,112,589 (11.42%)	4008 (31.59%)	1231 (24.31%)	2777 (36.41%)	< 0.001
Smoking status					< 0.001
Non-smoker	5,737,251 (58.91%)	7545 (59.46%)	3092 (61.07%)	4453 (58.39%)	
Ex-smoker	1,389,361 (14.27%)	2747 (21.65%)	976 (19.28%)	1771 (23.22%)	
Current-smoker	2,612,404 (26.82%)	2397 (18.89%)	995 (19.65%)	1402 (18.38%)	
Alcohol consumption					< 0.001
Non-drinker	4,923,347 (50.55%)	7864 (61.97%)	3063 (60.50%)	4801 (62.96%)	
Mild- to moderate-drinker	4,026,125 (41.34%)	3897 (30.71%)	1624 (32.08%)	2273 (29.81%)	
Heavy-drinker	789,544 (8.11%)	928 (7.31%)	376 (7.43%)	552 (7.24%)	
Regular physical activity	1,761,228 (18.08%)	2662 (20.98%)	1008 (19.91%)	1654 (21.69%)	< 0.001
Diabetes mellitus	791,392 (8.13%)	2104 (16.58%)	710 (14.02%)	1394 (18.28%)	< 0.001
Hypertension	2,443,461 (25.09%)	7288 (57.44%)	2450 (48.39%)	4838 (63.44%)	< 0.001
Dyslipidemia	1,676,784 (17.22%)	3914 (30.85%)	1422 (28.09%)	2492 (32.68%)	< 0.001
BMI (kg/m^2^)					< 0.001
BMI < 18.5	364,452 (3.74%)	368 (2.90%)	159 (3.14%)	209 (2.74%)	
18.5 ≤ BMI < 23	3,831,389 (39.34%)	4087 (32.21%)	1732 (34.21%)	2355 (30.88%)	
23 ≤ BMI < 25	2,396,612 (24.61%)	3264 (25.72%)	1289 (25.46%)	1975 (25.90%)	
25 ≤ BMI < 30	2,806,430 (28.82%)	4404 (34.71%)	1673 (33.04%)	2731 (35.81%)	
30 ≤ BMI	340,133 (3.49%)	566 (4.46%)	210 (4.15%)	356 (4.67%)	
CKD	908,905 (9.33%)	2244 (17.68%)	666 (13.15%)	1578 (20.69%)	< 0.001
WPW syndrome	836 (0.01%)	33 (0.26%)	4 (0.08%)	29 (0.38%)	< 0.001
Age (years)	46.38 ± 13.79	56.45 ± 13.59	54.00 ± 13.50	58.08 ± 13.41	< 0.001
Height (cm)	164.14 ± 9.20	162.92 ± 9.33	162.83 ± 9.18	162.97 ± 9.42	< 0.001
Weight (kg)	64.05 ± 11.67	64.60 ± 11.57	64.19 ± 11.59	64.87 ± 11.55	0.001
Systolic blood pressure (mmHg%)	122.22 ± 14.94	124.70 ± 15.45	123.68 ± 15.10	125.38 ± 15.65	< 0.001
Diastolic blood pressure (mmHg%)	76.25 ± 10.03	77.14 ± 10.04	76.97 ± 9.98	77.25 ± 10.09	< 0.001
Fasting glucose (mg/dL%)	96.93 ± 23.50	101.37 ± 26.87	100.26 ± 28.04	102.10 ± 26.04	< 0.001
Total cholesterol (mg/dL%)	195.31 ± 41.15	190.83 ± 44.26	193.89 ± 52.06	188.80 ± 38.09	< 0.001
High-density lipoprotein (mg/dL%)	56.56 ± 32.75	54.4 ± 31.90	54.82 ± 31.35	54.12 ± 32.27	< 0.001
eGFR (ml/min/1.73m^2^)	82.58 ± 42.49	76.92 ± 35.36	79.81 ± 37.07	75.00 ± 34.04	< 0.001

AF: atrial fibrillation; BMI: body mass index; CKD: chronic kidney disease; eGFR: estimated glomerular filtration rate; WPW syndrome: Wolff-Parkinson-White syndrome.

### Ventricular arrhythmias

**Figure 2:**
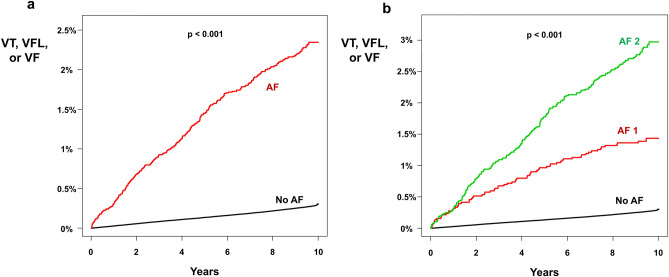
Risk of ventricular arrhythmia in people with AF. (**a**) Kaplan–Meier curve analysis (unadjusted) showed a significantly higher incidence of ventricular arrhythmia in the AF group. Multivariate adjusted HR was 4.593 (4.074–5.178). (**b**) The AF 1 group and AF 2 group had a significantly higher incidence of ventricular arrhythmia compared with the non-AF group. The unadjusted cumulative incidence of ventricular arrhythmia was significantly higher in the AF 2 group compared with the AF 1 group. After multivariate adjustment, AF 2 group had 1.639-fold higher risk of ventricular arrhythmia compared with AF 1 group. AF: atrial fibrillation; HR: hazard ratio; VF: ventricular fibrillation; VFL: ventricular flutter; VT: ventricular tachycardia.

During the follow-up period (until December 2018; 89,829,186 person-years of follow-up), a total of 25,581 ventricular arrhythmia (VT, VFL, and VF) events occurred. In the AF group, the incidence of ventricular arrhythmia was 2.472 (275 events during 111,248 person-years of follow-up), and it was 0.282 (25,306 events during 89,717,938 person-years of follow-up) in the non-AF group. The non-adjusted HR was 8.762 (95% CI 7.781–9.868; p < 0.001; Table 2). After adjusting for age, sex, BMI, smoking status, alcohol consumption status, physical activity status, hypertension, diabetes mellitus, and dyslipidemia, presence of new-onset AF was a significant risk factor for ventricular arrhythmia (HR = 4.593; 95% CI 4.074–5.178; p < 0.001; Table 2). The cumulative incidence of ventricular arrhythmia in the AF and non-AF groups is depicted in Fig. 2a and showed a significantly higher risk of ventricular arrhythmia in the AF group (log-rank p < 0.001).

**Table 2:** Incidence of ventricular arrhythmia in people with AF.

	n	Event number	Follow-up duration (person * years)	Incidence (per 1000 person * years)	Adjusted HR (Model 1)	Adjusted HR (Model 2)	Adjusted HR (Model 3)	Adjusted HR (Model 4)	Adjusted HR (Model 5)
No AF	9,739,016	25,306	89,717,938	0.282	1 (reference)	1 (reference)	1 (reference)	1 (reference)	1 (reference)
All AF (AF 1 + AF 2)	12,689	275	111,248	2.472	8.762 (7.781–9.868)	5.603 (4.975–6.31)	5.574 (4.948–6.279)	5.135 (4.558–5.785)	4.593 (4.074–5.178)
AF 1	5063	69	45,351	1.521	5.389 (4.255–6.825)	3.862 (3.049–4.891)	3.857 (3.045–4.885)	3.636 (2.871–4.606)	3.386 (2.673–4.290)
AF 2	7626	206	65,897	3.126	11.084 (9.664–12.713)	6.565 (5.722–7.531)	6.553 (5.712–7.518)	5.960 (5.194–6.838)	5.221 (4.546–5.996)

Ventricular arrhythmia is a composite of VT, VFL, and VF.

Incidence rate is per 1000 person-years of follow-up.

Model 1 is without multivariate adjustment.

Model 2 is adjusted for age and sex.

Model 3 is adjusted for Model 2 + BMI, smoking status, alcohol consumption, and physical activity.

Model 4 is adjusted for Model 3 + hypertension, diabetes, and dyslipidemia.

Model 5 is adjusted for Model 4 + chronic kidney disease, coronary artery disease, myocardial infarction, cardiomyopathy, and height.

Values are expressed as hazard ratio with 95% confidence interval.

AF: atrial fibrillation; BMI: body mass index; HR: hazard ratio; VF: ventricular fibrillation; VFL: ventricular flutter; VT: ventricular tachycardia.

The risk of ventricular arrhythmia differed between the non-AF group, the AF 1 group, and the AF 2 group, with an incidence of 0.282, 1.521 (69 events during 45,351 person-years of follow-up), and 3.126 (206 events during 65,897 person-years of follow-up), respectively. After multivariate adjustment, AF 1 and AF 2 were associated with a 3.386- and 5.221-fold increased risk of ventricular arrhythmia compared with the non-AF group, respectively (p < 0.001 for each; Table 2). The Kaplan–Meier curve analysis revealed a significantly higher cumulative incidence of ventricular arrhythmia events in the AF 2 group compared with the AF 1 group (log-rank p < 0.001; Fig. 2b; adjusted HR = 1.639; 95% CI 1.248–2.153; p < 0.001).

### Interaction analysis

The risk of ventricular arrhythmia in new-onset AF was increased by 5.679-fold higher (4.847–6.654) in people younger than 65 years and by 4.637-fold (3.871–5.556) higher in people 65 or older (p for interaction = 0.047; Table 3). A significant interaction was observed between AF and dyslipidemia, showing that people without dyslipidemia were at greater risk of ventricular arrhythmia due to AF (HR = 5.692 [4.924–6.579] vs. 4.256 [3.451–5.249]; p for interaction = 0.019; Table 3). No significant interactions were found between AF and sex, diabetes mellitus, hypertension, and CKD.

**Table 3:** Interaction analysis: non-AF vs. AF.

Subgroup	AF	n	Event	Duration (person * years)	Incidence (per 1000 person * years)	Adjusted HR (95% CI)	p for interaction
Age < 65 years	AF (–)	8,626,427	18,186	80,012,302	0.227	1 (reference)	0.047
	AF (+)	8681	155	78,895	1.965	5.679 (4.847–6.654)	
Age ≥ 65 years	AF (–)	1,112,589	7120	9,705,636	0.734	1 (reference)	
	AF (+)	4008	120	32,353	3.709	4.637 (3.871–5.556)	
Male	AF (–)	5,396,129	14,254	49,483,358	0.288	1 (reference)	0.121
	AF (+)	7478	177	64,558	2.742	5.332 (4.595–6.187)	
Female	AF (–)	4,342,887	11,052	40,234,580	0.275	1 (reference)	
	AF (+)	5211	98	46,690	2.099	4.733 (3.878–5.776)	
Diabetes mellitus (–)	AF (–)	8,947,624	21,673	82,630,564	0.262	1 (reference)	0.928
	AF (+)	10,585	215	93,790	2.292	5.104 (4.461–5.840)	
Diabetes mellitus (+)	AF (–)	791,392	3633	7,087,374	0.513	1 (reference)	
	AF (+)	2104	60	17,458	3.437	5.279 (4.088–6.817)	
Hypertension (–)	AF (–)	7,295,555	13,937	67,548,850	0.206	1 (reference)	0.898
	AF (+)	5401	74	48,636	1.522	5.034 (4.005–6.327)	
Hypertension (+)	AF (–)	2,443,461	11,369	22,169,088	0.513	1 (reference)	
	AF (+)	7288	201	62,612	3.210	5.245 (4.561–6.031)	
Dyslipidemia (–)	AF (–)	8,062,232	18,593	74,313,814	0.250	1 (reference)	0.019
	AF (+)	8775	186	76,943	2.417	5.692 (4.924–6.579)	
Dyslipidemia (+)	AF (–)	1,676,784	6713	15,404,124	0.436	1 (reference)	
	AF (+)	3914	89	34,305	2.594	4.256 (3.451–5.249)	
CKD (–)	AF (–)	8,830,111	21,723	81,466,280	0.267	1 (reference)	0.822
	AF (+)	10,445	210	92,519	2.270	5.070 (4.425–5.811)	
CKD (+)	AF (–)	908,905	3583	8,251,658	0.434	1 (reference)	
	AF (+)	2244	65	18,729	3.471	5.396 (4.219–6.901)	

Hazard ratios are adjusted for age, sex, BMI, smoking status, alcohol consumption, physical activity, hypertension, diabetes, and dyslipidemia.

AF: atrial fibrillation; BMI: body mass index; CI: confidence interval; CKD: chronic kidney disease; HR: hazard ratio.

A similar pattern of interaction was observed when AF was subdivided into AF 1 and AF 2. Regarding the risk of ventricular arrhythmia, age and dyslipidemia showed significant interactions with AF, and young people and those without dyslipidemia had a significantly increased risk of developing ventricular arrhythmia attributable to AF (Table 4).

**Table 4:** Interaction analysis: non-AF vs. AF 1 vs. AF 2.

Subgroup	AF	n	Event	Duration (person * years)	Incidence (per 1000 person * years)	Adjusted HR (95% CI)	p for interaction
Age < 65 years	No AF	8,626,427	18,186	80,012,302	0.227	1 (reference)	< 0.001
	AF 1	3832	34	35,215	0.965	3.059 (2.185–4.284)	
	AF 2	4849	121	43,679	2.770	7.500 (6.271–8.970)	
Age ≥ 65 years	No AF	1,112,589	7120	9,705,636	0.734	1 (reference)	
	AF 1	1231	35	10,136	3.453	4.453 (3.194–6.208)	
	AF 2	2777	85	22,218	3.826	4.718 (3.808–5.845)	
Male	No AF	5,396,129	14,254	49,483,358	0.288	1 (reference)	0.315
	AF 1	2823	41	24,944	1.644	3.645 (2.682–4.953)	
	AF 2	4655	136	39,614	3.433	6.199 (5.233–7.343)	
Female	No AF	4,342,887	11,052	40,234,580	0.275	1 (reference)	
	AF 1	2240	28	20,407	1.372	3.578 (2.469–5.185)	
	AF 2	2971	70	26,283	2.663	5.427 (4.288–6.868)	
Diabetes mellitus (–)	No AF	8,947,624	21,673	82,630,564	0.262	1 (reference)	0.658
	AF 1	4353	53	39,366	1.346	3.466 (2.647–4.539)	
	AF 2	6232	162	54,424	2.977	6.040 (5.173–7.053)	
Diabetes mellitus ( +)	No AF	791,392	3633	7,087,374	0.513	1 (reference)	
	AF 1	710	16	5985	2.673	4.389 (2.685–7.174)	
	AF 2	1394	44	11,473	3.835	5.701 (4.232–7.679)	
Hypertension (–)	No AF	7,295,555	13,937	67,548,850	0.206	1 (reference)	0.840
	AF 1	2613	25	23,879	1.047	3.785 (2.557–5.604)	
	AF 2	2788	49	24,757	1.979	6.050 (4.569–8.012)	
Hypertension ( +)	No AF	2,443,461	11,369	22,169,088	0.513	1 (reference)	
	AF 1	2450	44	21,472	2.049	3.565 (2.652–4.794)	
	AF 2	4838	157	41,140	3.816	6.036 (5.155–7.067)	
Dyslipidemia (–)	No AF	8,062,232	18,593	74,313,814	0.250	1 (reference)	0.043
	AF 1	3641	47	32,707	1.437	3.908 (2.935–5.203)	
	AF 2	5134	139	44,237	3.142	6.734 (5.696–7.960)	
Dyslipidemia ( +)	No AF	1,676,784	6713	15,404,124	0.436	1 (reference)	
	AF 1	1422	22	12,645	1.740	3.153 (2.074–4.792)	
	AF 2	2492	67	21,660	3.093	4.809 (3.778–6.121)	
CKD (–)	No AF	8,830,111	21,723	81,466,280	0.267	1 (reference)	0.790
	AF 1	4397	58	39,644	1.463	3.755 (2.901–4.859)	
	AF 2	6048	152	52,875	2.875	5.855 (4.990–6.871)	
CKD ( +)	No AF	908,905	3583	8,251,658	0.434	1 (reference)	
	AF 1	666	11	5707	1.927	3.138 (1.736–5.673)	
	AF 2	1578	54	13,022	4.147	6.334 (4.839–8.291)	

Hazard ratios are adjusted for age, sex, BMI, smoking status, alcohol consumption, physical activity, hypertension, diabetes, and dyslipidemia.

AF: atrial fibrillation; BMI: body mass index; CI: confidence interval; CKD: chronic kidney disease; HR: hazard ratio.

### Simplified exclusion criteria

We performed additional analysis with a simplified exclusion criteria: age < 20 years and prior history of ventricular arrhythmia (VT, VFL, and VF) during screening period (2002 to 2008) (Supplementary Fig. S1). With this cohort, 46,082 AF patients were identified in 2009 and had 6.395-fold higher risk of lethal ventricular tachycardia compared with people without AF after multivariate adjustment (95% CI 6.082–6.725; p < 0.001; Supplementary Table S2). Kaplan–Meier curve analysis showed significant and continuous divergence of cumulative incidence of lethal ventricular arrhythmia (log-rank p < 0.001; Supplementary Fig. S2). Since this cohort with simplified exclusion criteria included people with heart failure, PVC, and prior diagnosis of AF (thus longer exposure to deleterious effect of AF), the incidence of lethal ventricular arrhythmia was significantly higher as compared with our main cohort (incidence = 4.423 vs. 2.472; p < 0.001).

## Discussion

This study demonstrates the chronological association between new-onset AF and ventricular arrhythmia. People who were diagnosed with AF in 2009 were at 4.593-fold increased risk of suffering VT, VFL, or VF compared with people without AF during 10 years of follow-up. The risk was even higher in the AF 2 group, which demonstrated a 5.221-fold higher risk of ventricular arrhythmia. Since we excluded people with prior history of AF, VT, VFL, and VF and had a sufficient duration of clinical follow-up, the chronological influence of new-onset AF (in 2009) on ventricular arrhythmia was evaluated in our study. Based on large sample size, we performed subgroup analyses and revealed that age and dyslipidemia had significant interactions with new-onset AF regarding development of ventricular arrhythmia.

### Underlying mechanism

Physiologic conduction delays in the atrioventricular node and the atrioventricular nodal effective refractory period prevent rapid conduction of atrial signals, especially during AF, to the ventricles^15,16^. Furthermore, obligatory atrioventricular delay prevents atrial signal conduction to the ventricles during the vulnerable period. Despite these protective roles of physiologic conduction delay by the atrioventricular node, it is possible that rapid ventricular activation by AF might create conditions that enable the occurrence of ventricular arrhythmias. Under high adrenergic activation situations such as physical exercise, atrioventricular delay and atrioventricular nodal effective refractive period can progressively shorten, allowing rapid activation of ventricles and possibly the R-on-T phenomenon^15^. Prior study actually reported a R-on-T phenomenon in AF patients which was recorded in implantable cardioverter defibrillator suggesting that AF can be an atypical trigger of lethal ventricular arrhythmias even in patients without pre-excitation^17^. Accompanied pre-excitation in AF patients is itself a significant risk factor for sudden death and inappropriate synchronization during direct current cardioversion can be an another potential cause of AF degenerating into VF^18^.

Sympathetic denervation has shown to relieve electrical storm suggesting sympathetic activation may play an important role in occurrence of ventricular fibrillation^19–21^ and prior studies demonstrated that sympathetic nervous system is activated in AF patients and has critical role in AF development^22^. Furthermore, AF is associated with significant patient discomfort which might activate sympathetic tone^2,23^. Activation of sympathetic nervous system might be another possible underlying mechanism linking AF with lethal ventricular arrhythmias.

Atrial myopathy is a potential underlying pathophysiology of AF characterized by fibrofatty infiltration and loss of sarcomeres^24^. The underlying cause of atrial myopathy is not clear, but aging, increased pressure of the left atrium, inflammation, and genetic predisposition are proposed mechanisms^24–26^. These pathologic processes might induce myopathy not only in the atrium, but also in the ventricles, predisposing patients to development of ventricular arrhythmias. A common underlying myopathy in both the atrium and the ventricle might explain the significantly increased risk of ventricular arrhythmia in people with AF a phenomenon observed in this study.

Genetic variations can also play a role in co-existence of AF and lethal ventricular arrhythmias. Prior study has reported a phenomenon of new-onset AF as the first clinical manifestation of Brugada syndrome, a genetic disorder predisposing to significantly increased of lethal ventricular arrhythmia^27^. Genetic variations in SCN5A and KCNE5 gene have been suggested as a potential underlying link between AF and Brugada syndrome^28–30^. It is possible that various genetic variations associated with channelopathy or cardiomyopathy can affect both atrium and ventricle causing both AF and lethal ventricular arrhythmias.

### Crosstalk between atrial and ventricular arrhythmia

We previously reported an increased risk of AF in people with PVC^31^. Since the atria and ventricles are electrically connected to each other by the atrioventricular node and His bundle, arrhythmias arising from the atrium can influence the ventricle, and vice versa. The atrium and ventricle are also mechanically correlated. For example, a decrease in left ventricular systolic function due to AF and subsequent development of heart failure can provoke ventricular arrhythmias. Frequent PVCs can also depress left ventricular ejection fraction, which can elevate left atrial pressure and create a favorable environment for AF as shown in our previous study^31^.

In patients with implantable cardioverter-defibrillators for secondary prevention, presence of AF was associated with an increased risk of lethal ventricular arrhythmias in a prior study^32^. Our study is in accordance with the study in a more general AF population. Presence of AF in patients presenting with lethal ventricular arrhythmia was associated with a significantly increased mortality probably due to progressive worsening of heart function, increased shocks from implantable cardioverter-defibrillators, systematic inflammation, and heterogeneous atrioventricular conduction^33^. Such interactions between atrial and ventricular arrhythmias will be a valuable area of future research.

Whether radiofrequency catheter ablation (RFCA) of AF can reduce the risk of ventricular arrhythmia is of potential interest. In patients with AF and advanced heart failure, RFCA improves left ventricular ejection fraction and clinical outcomes such as hospitalization for heart failure and overall mortality^34^. By improving ventricular function and ganglionic plexus modulation, RFCA might have the potential to reduce lethal ventricular arrhythmias in people with AF, a hypothesis that should be tested in future trials.

### Limitations

This study has several limitations. First, there might be inaccuracies in coding, missing data, and selection bias since this was a retrospective study based on data stored in a nationwide health insurance organization. However, our strategy to identify various diseases such as AF, stroke, heart failure, hypertension, or diabetes mellitus has been validated in multiple prior studies^13,14,35,36^. Second, our study was exclusively based on an East Asian population, and caution is needed when applying our results to other ethnic groups. Third, genetic evaluation such as whole exon sequencing, which might reveal common pathogenic variants responsible for both AF and ventricular arrhythmia, was not available in this study.

## Conclusions

New-onset AF was closely associated with a higher incidence of ventricular arrhythmia, a composite of VT, VFL, and VF. The potential risk of lethal ventricular arrhythmias in people with AF should be kept in mind in real-world clinical practice.

## Supplementary Information

Supplementary Information.

## Abbreviations

AF: Atrial fibrillation
BMI: Body mass index
HR: Hazard ratio
ICD: International classification of disease
K-NHIS: Korean National Health Insurance Service
PVC: Premature ventricular contraction
VF: Ventricular fibrillation
VFL: Ventricular flutter
VT: Ventricular tachycardia
