# Supplementary material for: Atrial fibrillation is associated with increased risk of lethal ventricular arrhythmias
Source: Sci Rep. 2021 Sep 13;11:18111. doi: 10.1038/s41598-021-97335-y (PMC8438063; doi:10.1038/s41598-021-97335-y)
Supplement: Supplementary file 1 — Supplementary Information. [file 41598_2021_97335_MOESM1_ESM.docx]

**Atrial Fibrillation is Associated with Increased Risk of Lethal Ventricular Arrhythmias**

Yun Gi Kim,^1^ Yun Young Choi,^1^ Kyung-Do Han,^2^ Kyongjin Min,^1^ Ha Young Choi,^1^ Jaemin Shim,^1^ Jong-Il Choi,^1^* and Young-Hoon Kim^1^

^1^Division of Cardiology, Department of Internal Medicine, Korea University College of Medicine and Korea University Anam Hospital, Seoul, Republic of Korea

^2^Department of Statistics and Actuarial Science, Soongsil University, Seoul, Republic of Korea

*Address for correspondence: Jong-Il Choi, MD, PhD, MHSc

^1^Division of Cardiology, Department of Internal Medicine, Korea University College of Medicine and Korea University Anam Hospital, Seoul, Republic of Korea

73 Goryeodae-ro, Seongbuk-gu, Seoul 02841, Republic of Korea

Tel: 82-2-920-5445

Fax: 82-2-927-1478

E-mail: [jongilchoi@korea.ac.kr](mailto:jongilchoi@korea.ac.kr)

**Running title:** Atrial fibrillation and lethal ventricular arrhythmias

**Disclosure:** The authors have nothing to disclose.

**Total word count:** 5,978

The first two authors contributed equally to this work.

**Supplementary Table S1.** Diagnostic codes.

|  | ICD-10 codes |
| --- | --- |
| **Premature ventricular contraction** | I49.3 |
| **Heart failure** | I50 (all sub-codes) |
| **Ventricular arrhythmia composite** | I47.2, I49.0 |
| **Ventricular tachycardia** | I47.2 (all sub-codes) |
| **Ventricular fibrillation and flutter** | I49.0 |
| **Atrial fibrillation** | I48 (all sub-codes) |
| **Type 2 diabetes** | E11 – E14 (all sub-codes) |
| **Hypertension** | I10 – I13, I15 (all sub-codes) |
| **Dyslipidemia** | I78 (all sub-codes) |
| **Chronic kidney disease** | Based on creatinine checked during national health check-up |
| **Stroke** | I63, I64 (all sub-codes) |


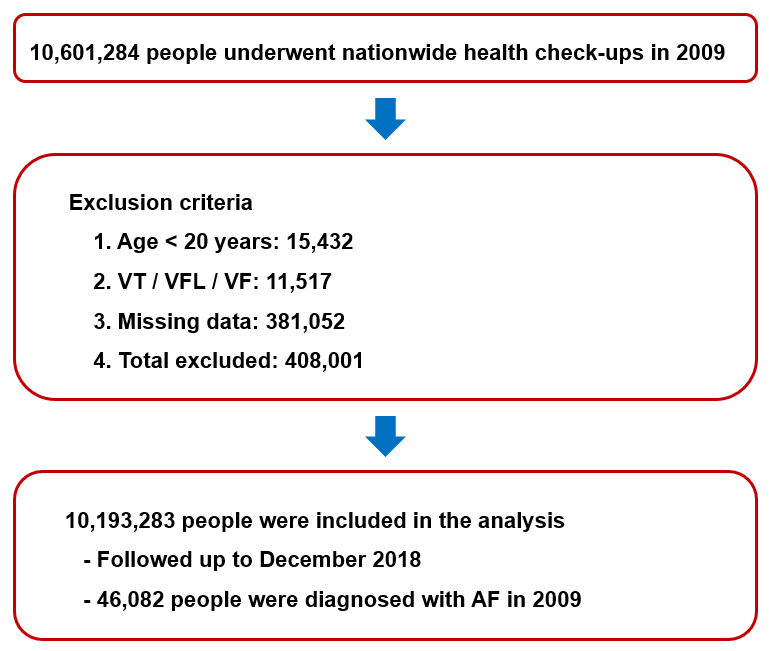


**Supplementary Figure S1.** Study flow (simplified exclusion criteria).

AF: atrial fibrillation; VF: ventricular fibrillation; VFL: ventricular flutter; VT: ventricular tachycardia.


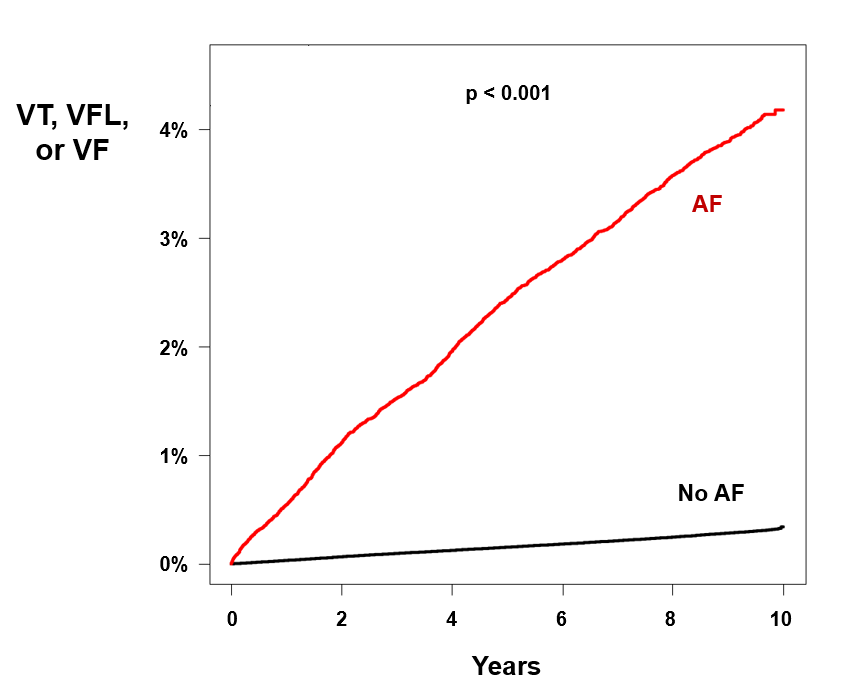


**Supplementary Figure S2.** Risk of ventricular arrhythmia in people with AF (simplified exclusion criteria).

Kaplan-Meier curve analysis (unadjusted) showed a significantly higher incidence of ventricular arrhythmia in the AF group. Multivariate adjusted HR was 6.395 (95% confidence interval = 6.082 – 6.725; p < 0.001).

**Supplementary Table S2.** Incidence of ventricular arrhythmia in people with AF (simplified exclusion criteria).

|  | **n** | **Event**  **number** | **Follow-up**  **duration**  **(person*years)** | **Incidence**  **(per 1,000 person*years)** | **Adjusted HR**  **(Model 1)** | **Adjusted HR**  **(Model 2)** | **Adjusted HR**  **(Model 3)** | **Adjusted HR**  **(Model 4)** |
| --- | --- | --- | --- | --- | --- | --- | --- | --- |
| No AF | 10,147,201 | 29,554 | 93,241,755 | 0.317 | 1 (reference) | 1 (reference) | 1 (reference) | 1 (reference) |
| AF 2 | 46,082 | 1,687 | 381,443 | 4.423 | 13.95 0 (13.282 – 14.652) | 7.129 (6.782 – 7.494) | 7.117 (6.771 – 7.482) | 6.395 (6.082 – 6.725) |

Ventricular arrhythmia is a composite of VT, VFL, and VF.

Incidence rate is per 1,000 person-years of follow-up.

Model 1 is without multivariate adjustment.

Model 2 is adjusted for age and sex.

Model 3 is adjusted for Model 2 + BMI, smoking status, alcohol consumption, and physical activity.

Model 4 is adjusted for Model 3 + hypertension, diabetes, and dyslipidemia.

Values are expressed as hazard ratio with 95% confidence interval.

AF: atrial fibrillation; BMI: body mass index; HR: hazard ratio; VF: ventricular fibrillation; VFL: ventricular flutter; VT: ventricular tachycardia.
